# Supplementary material for: Multi‐dataset identification of innovative feature genes and molecular mechanisms in keratoconus
Source: J Cell Mol Med. 2024 Sep 19;28(18):e70079. doi: 10.1111/jcmm.70079 (PMC11412914; doi:10.1111/jcmm.70079)
Supplement: Supplementary file 2 — Material S2. [file JCMM-28-e70079-s002.zip › 7.GSEA analysis/1.ARL11/KEGG_PROTEIN_EXPORT.html]

Details for gene set KEGG\_PROTEIN\_EXPORT[GSEA]

|  || Dataset | ARL11.group.cls#h\_versus\_l |
| Phenotype | group.cls#h\_versus\_l |
| Upregulated in class | h |
| GeneSet | KEGG\_PROTEIN\_EXPORT |
| Enrichment Score (ES) | 0.25157923 |
| Normalized Enrichment Score (NES) | 0.8102075 |
| Nominal p-value | 0.8180039 |
| FDR q-value | 1.0 |
| FWER p-Value | 1.0 |
Table: GSEA Results Summary

  

Fig 1: Enrichment plot: KEGG\_PROTEIN\_EXPORT      
 Profile of the Running ES Score & Positions of GeneSet Members on the Rank Ordered List

  

| SYMBOL | TITLE | RANK IN GENE LIST | RANK METRIC SCORE | RUNNING ES | CORE ENRICHMENT || 1 | SEC61A2 | na | 2129 | 0.287 | 0.2516 | Yes |
| 2 | SRP54 | na | 13344 | 0.024 | -0.0452 | No |
| 3 | SRP9 | na | 13828 | 0.020 | -0.0371 | No |
| 4 | IMMP1L | na | 13969 | 0.019 | -0.0205 | No |
| 5 | SRP19 | na | 14590 | 0.014 | -0.0227 | No |
| 6 | SRP14 | na | 16107 | 0.003 | -0.0632 | No |
| 7 | SEC11A | na | 16344 | 0.001 | -0.0685 | No |
| 8 | SEC61B | na | 16352 | 0.001 | -0.0672 | No |
| 9 | HSPA5 | na | 18392 | -0.013 | -0.1119 | No |
| 10 | SPCS2 | na | 18859 | -0.016 | -0.1078 | No |
| 11 | SPCS1 | na | 19096 | -0.018 | -0.0955 | No |
| 12 | SEC11C | na | 19139 | -0.018 | -0.0773 | No |
| 13 | SRP72 | na | 20302 | -0.026 | -0.0824 | No |
| 14 | SRPRB | na | 20933 | -0.031 | -0.0671 | No |
| 15 | SRP68 | na | 21000 | -0.031 | -0.0348 | No |
| 16 | IMMP2L | na | 21554 | -0.036 | -0.0120 | No |
| 17 | SEC63 | na | 22358 | -0.044 | 0.0124 | No |
| 18 | SEC61G | na | 22550 | -0.046 | 0.0566 | No |
| 19 | SEC61A1 | na | 22654 | -0.047 | 0.1045 | No |
| 20 | OXA1L | na | 23475 | -0.055 | 0.1413 | No |
| 21 | SPCS3 | na | 24089 | -0.064 | 0.1929 | No |
| 22 | SEC62 | na | 26808 | -0.104 | 0.2282 | No |
Table: GSEA details [plain text format]

  

Fig 2: KEGG\_PROTEIN\_EXPORT      
 Blue-Pink O' Gram in the Space of the Analyzed GeneSet

  

Fig 3: KEGG\_PROTEIN\_EXPORT: Random ES distribution      
 Gene set null distribution of ES for **KEGG\_PROTEIN\_EXPORT**
